# Supplementary material for: The genomic and transcriptional landscape of primary central nervous system lymphoma
Source: Nat Commun. 2022 May 10;13:2558. doi: 10.1038/s41467-022-30050-y (PMC9091224; doi:10.1038/s41467-022-30050-y)
Supplement: Supplementary file 1 — Supplementary Information [file 41467_2022_30050_MOESM1_ESM.pdf]

Supplementary Information

1. Supplementary figures 1-10 with figure legends
2. Supplementary Reference

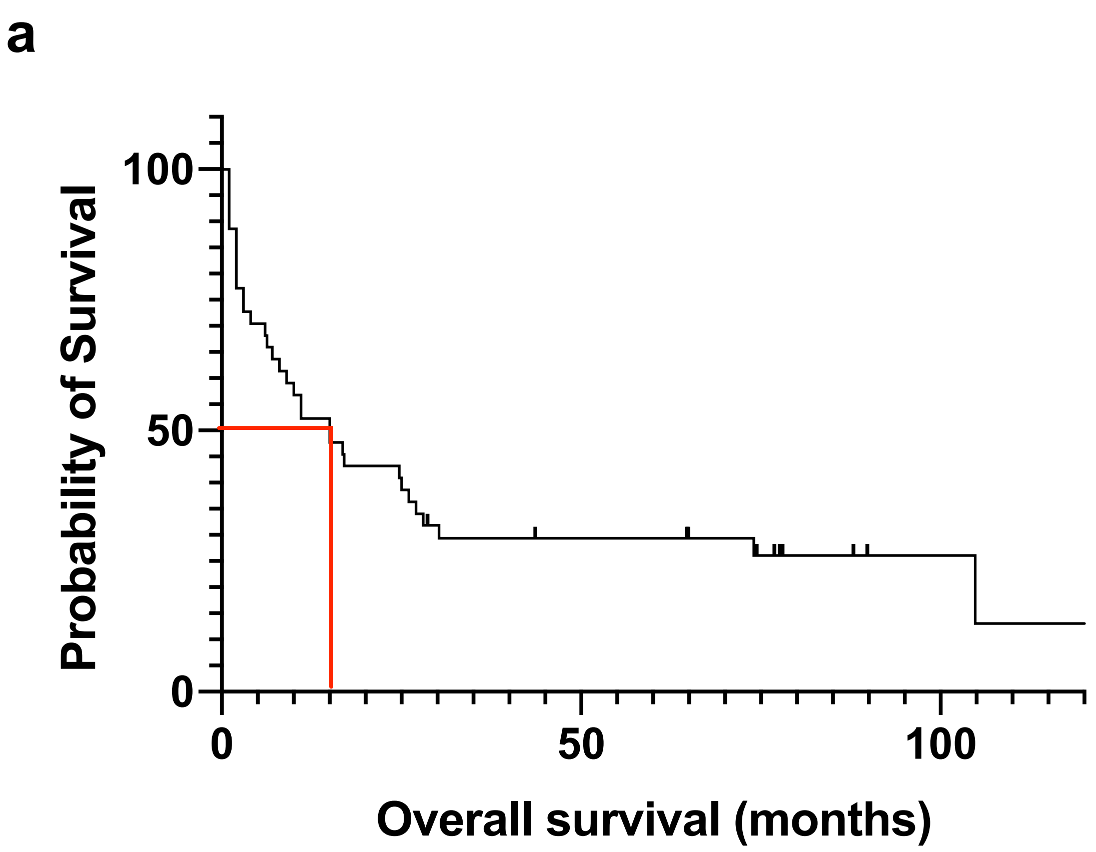

Number of rows	51
# of blank lines	7
# rows with impossible data	0
# censored subjects	11
# deaths/events	33
Median survival	15

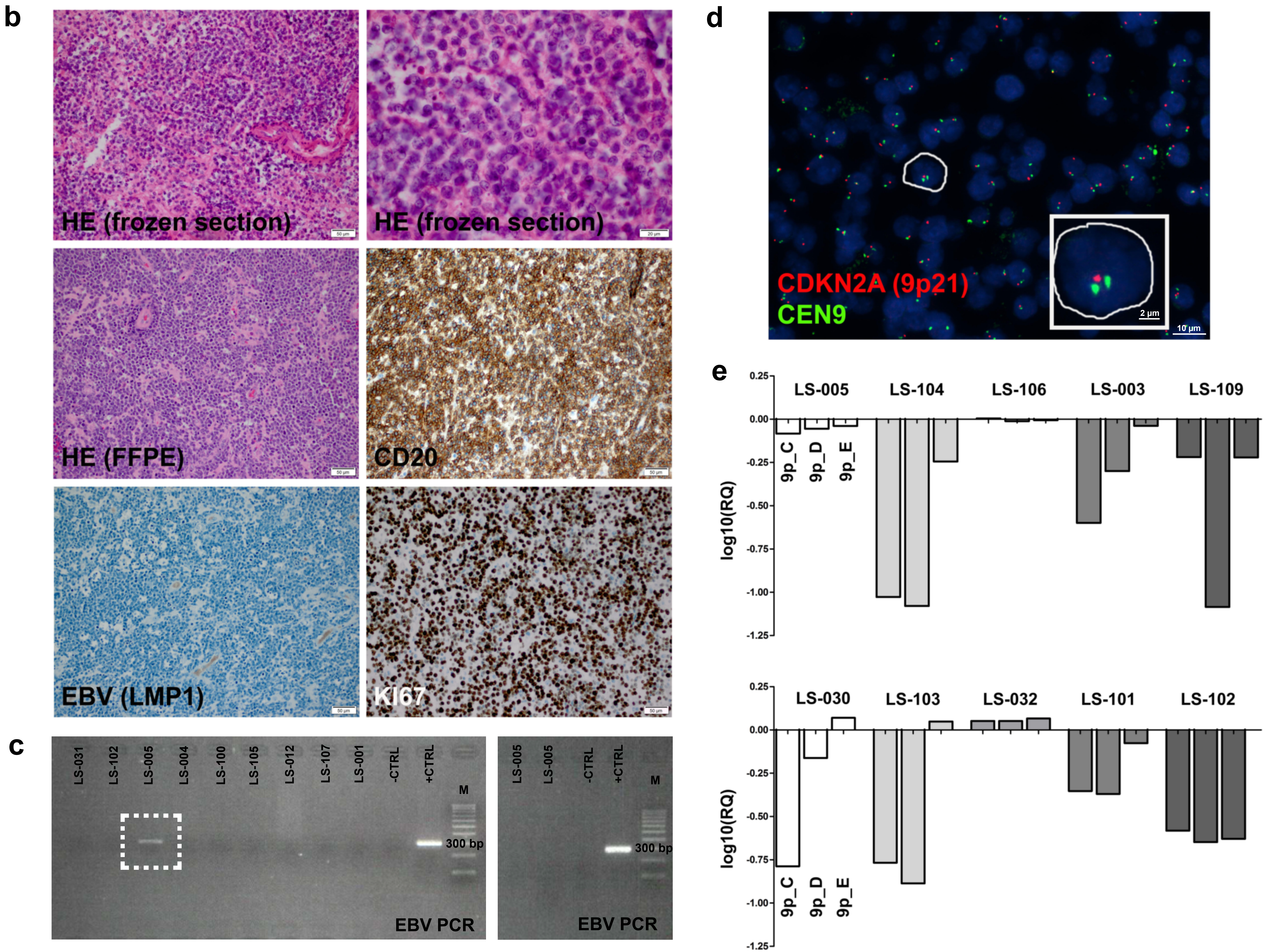

Supplementary figure 1

Supplementary figure 1: Characterisation of CNSL samples.

(a) Kaplan-Meier survival curve analysis (GraphPad Prism 9, version 9.0.0) for the CNSL cohort. Follow up data was available for 44 patients. The follow up time ranged from one to 104 months with a median survival of 15.0 months (red line). Censored subjects are indicated on the Kaplan-Meier curve as tick marks. Source data are provided as a Source Data file. (b) Histological and immunohistological evaluation of primary CNS lymphoma samples. Hematoxylin & Eosin (H&E) was used to stain frozen sections for histological analysis of intraoperative tissue specimens to evaluate tumour cell content and tissue quality. Formalin-fixed paraffin-embedded (FFPE) samples showed a dense infiltrate of malignant, abnormal large lymphoid cells with vesicular chromatin, prominent nucleoli and cuffing of the capillary vessels. The diagnosis was confirmed by positive immunohistochemistry for CD20. The latent membrane protein 1 (LMP1) of Epstein-Barr virus was not expressed and Ki67 showed very high proliferative activity (> 80%) of the lymphoma cells. (c) Detection of Epstein-Barr virus (EBV) DNA in CNSL specimens by PCR targeting a highly conserved region of the EBNA-1 (BKRF1) gene (297 bp, upper panel). Sample LS-GD-005 was positive in one PCR run (left, black square) but negative in a repeated run (right). M (1 kb DNA ladder), +CTRL (positive control), -CTRL (negative control; water). Source data are provided as a Source Data file. (d) Exemplary result of CNV validation experiments by fluorescence in situ hybridization (FISH). FISH analysis with a p16 (*CDKN2A*) and CEN9 probe revealed heterozygous deletion with loss of one of the red (*CDKN2A*) signals in a nucleus and preservation of the two centromeric green (CEN9) signals (patient LS-031). (e) Quantitative RT-PCR demonstrated homozygous deletion of *CDKN2A* in patients LS-104, LS-003, LS-109, LS-030, LS-103, LS-101, and LS-102 and no *CDKN2A* deletion in patients LS-005, LS-106, and LS-032 using three different primer (9p21.3_C, _D, _E) for the region of *CDKN2A*. Source data are provided as a Source Data file.

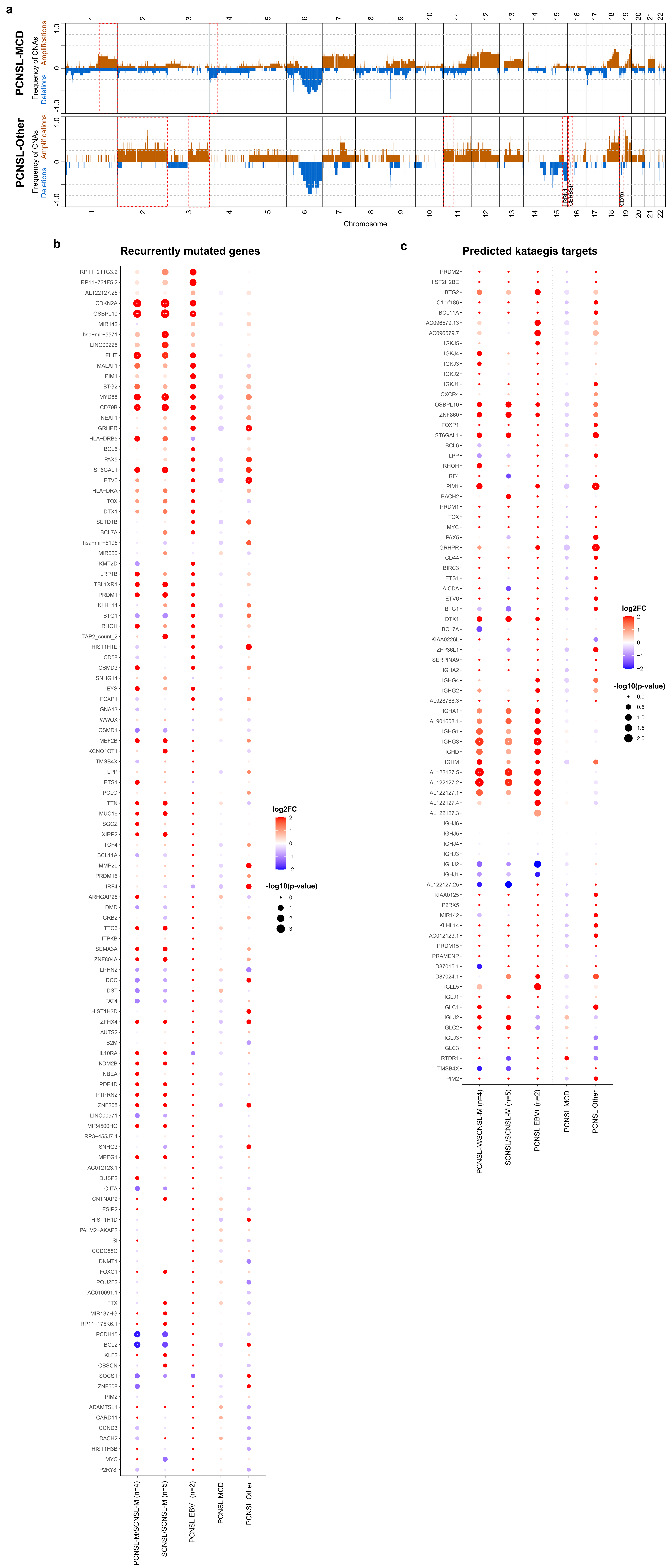

Supplementary figure 2

Supplementary figure 2: Genomic structural variation in PCNSL-MCD and PCNSL-Other.

(a) Relative prevalence of somatic copy number aberrations in tumour samples (middle panel), showing presence of at least one copy number gain (orange bars), copy number loss (blue bars), as a proportion of analysed samples. The differences between PCNSL-MCD and PCNSL-Other are highlighted in red and some candidate genes detected to be significant by Gistic2 (q-value <0.25) are shown. PCNSL-Other demonstrated significantly more deletions in *CREBBP* (p=0.04648, One-tailed Mann-Whitney U test, not corrected for multiple testing) compared to PCSNL-MCD. (b, c) The dot plots show the log2 fold change (colour) and significance (size of dot) of alteration frequencies of genes in PCNSL compared to different subcohorts, PCNSL-MCD, and PCNSL-Other.

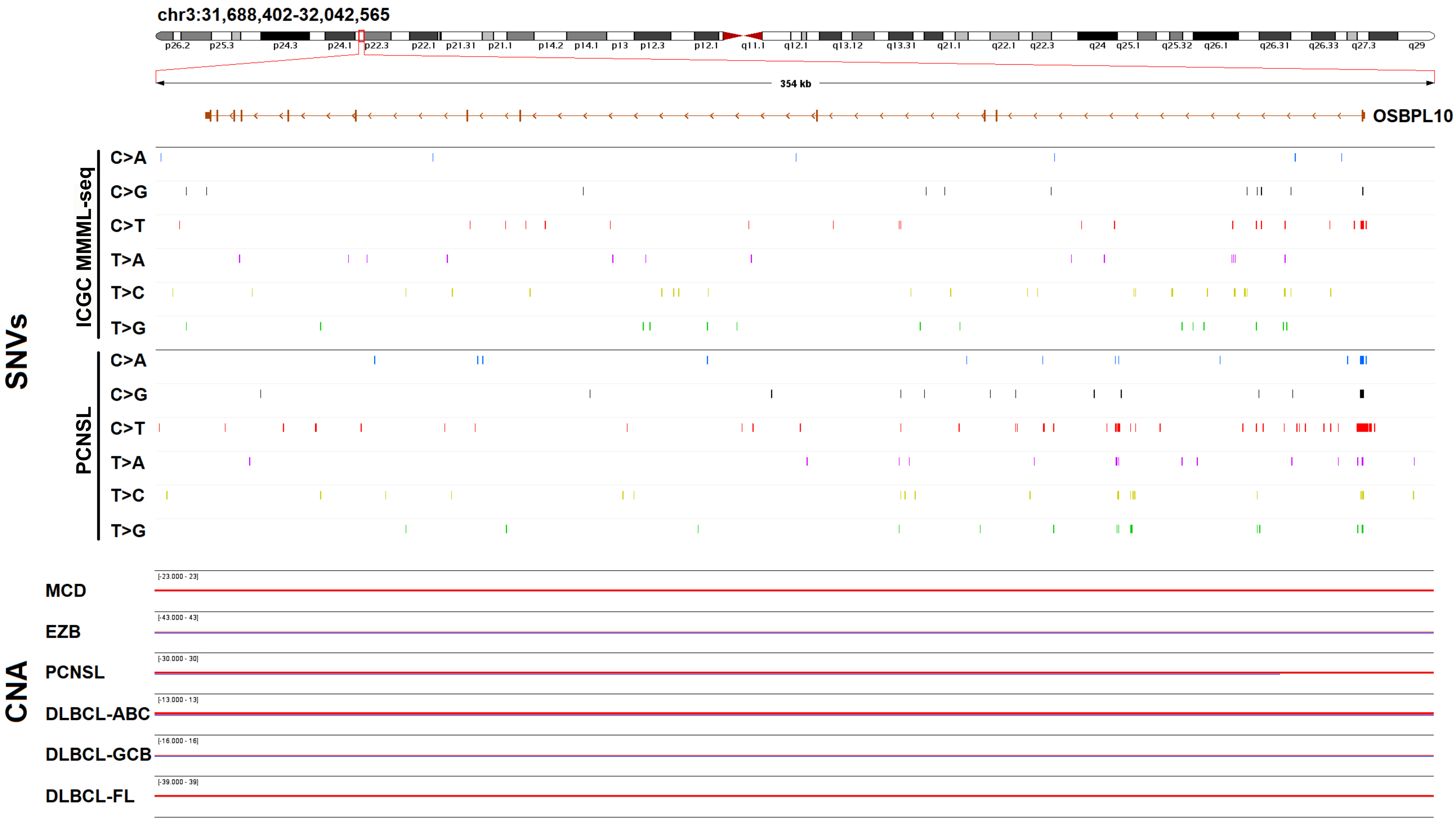

Supplementary figure 3

Supplementary figure 3: Somatic SNV and CNA landscape around OSBPL10.

Somatic single nucleotide variants (SNVs) from samples in our study indicate enrichment of C>T mutations in the first exon of the OSBPL10 gene, consistent with aberrant somatic hypermutation (aSHM) in previous studies. For the SNV panel, each somatic mutation observed in a sample is marked, and at this resolution, the clustered mutations in the OSBPL10 promoter appear as a block. In-depth breakdown of mutational frequencies between stratified subcohorts are shown in dot plots in the manuscript (Figure 2 b). The copy number alterations (CNAs) show that very few samples are affected by copy number changes irrespective of cohort stratification. The CNA panels are normalized to the stratified sub-cohort size, with red indicating cumulative amplifications in samples, and blue indicating cumulative deletions.

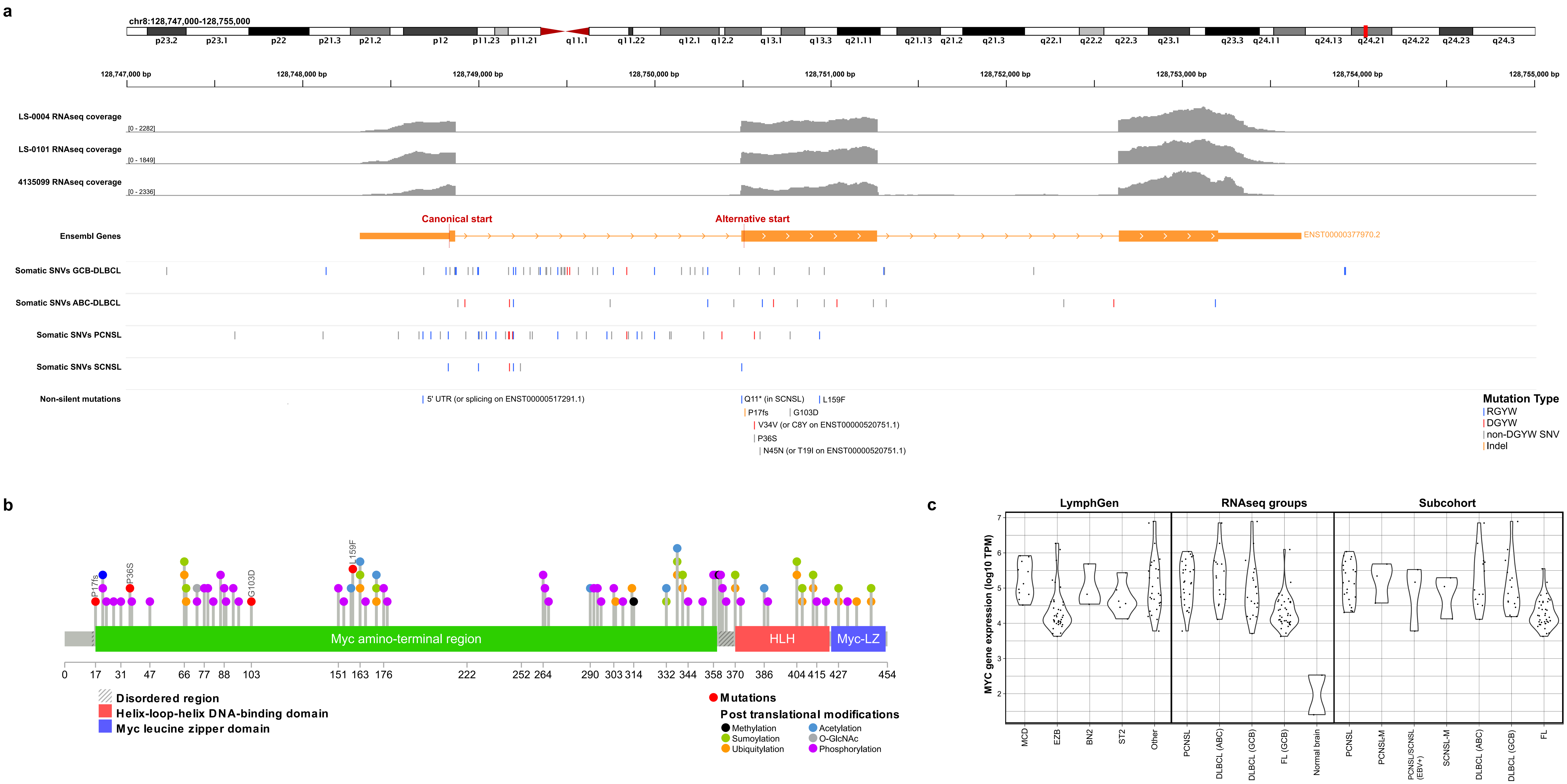

Supplementary figure 4

Supplementary figure 4: Mutational analysis on MYC gene and protein.

(a) Raw RNAseq reads aligning across the *MYC* transcript in two PCNSL (LS-0004, LS-0101) and one ABC-DLBCL (4135099, upper panel). The gene model for the canonical transcript for *MYC*, ENST00000377970.2, marking alternative start sites that encode for the proteins P01106-1 and P01106-2. Distribution of somatic SNVs and indels in *MYC* (lower panel). Mutations which affect the RGYW/DGYW motifs, are indicated by blue and red dots; grey dots show mutations outside the motifs. Somatic SNVs and indels identified in PCNSL and SNCSL samples which may cause potential protein coding changes on either the canonical or other transcripts of *MYC*. (b) The lollipop plot shows the protein domains (coloured boxes) over protein coding positions of the MYC protein isoform P01106-2 encoded by ENST00000377970.2 (grey bar). The somatic non-silent point mutations observed in the PCNSL samples that mapped to the MYC isoform are shown as red dots, with the protein coding changes annotated above the dot. Each somatic mutation was only observed once in the series. The post translational modification sites from PhosphoSitePlus v6.6.0.2 are shown as other coloured dots. The height of the dots does not encode for recurrence. (c) The violin plots show the log10 TPM RNA expression of *MYC* in the different subgroups.

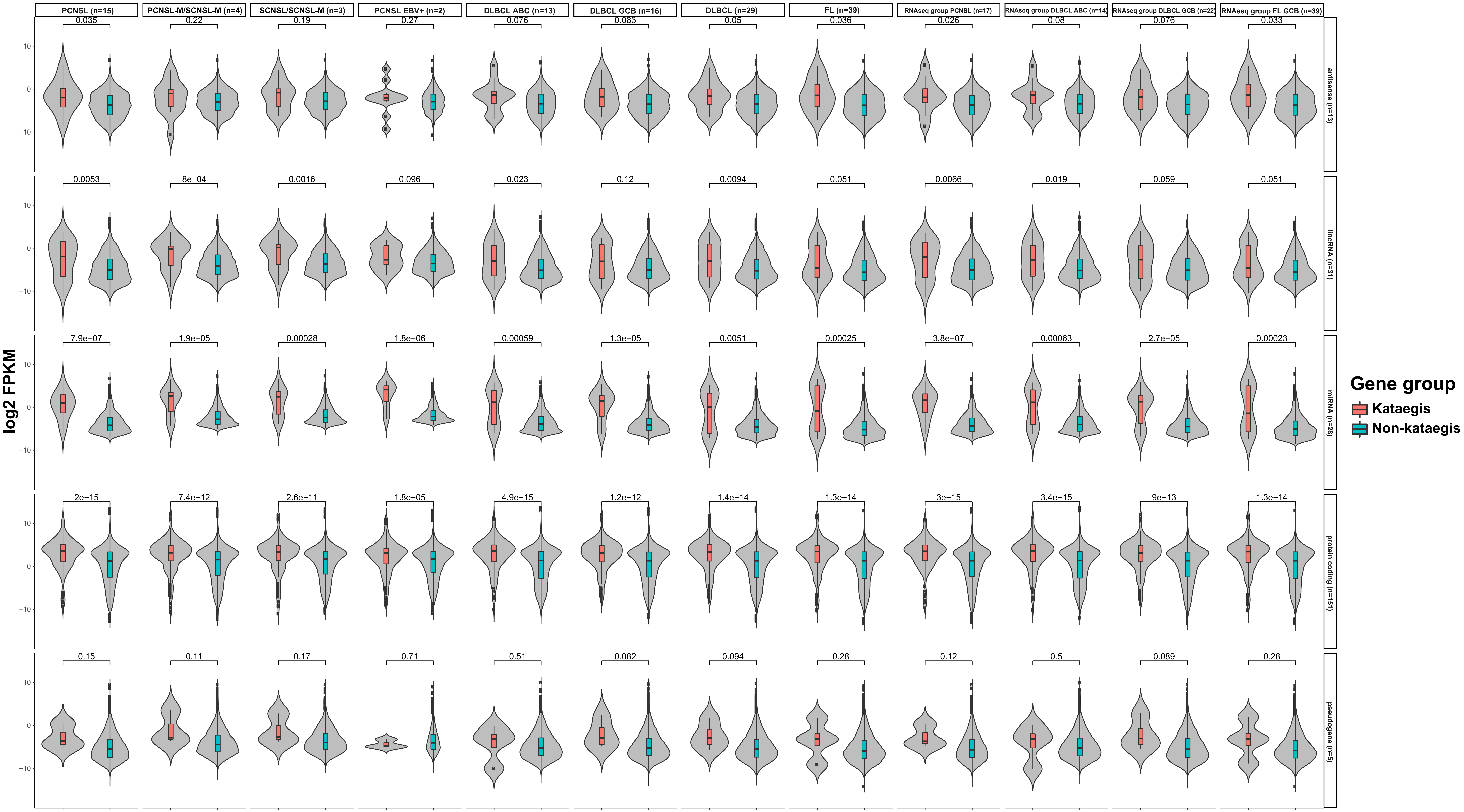

Supplementary figure 5

Supplementary figure 5: Kataegis events in PCNSL.

The violin plot shows the RNA expression of genes with kataegis loci compared to those without for antisense, long non-coding RNA, miRNA and protein coding genes for all subcohorts and RNA subgroups (one-sided Wilcoxon rank sum test not corrected for multiple testing). Box and whisker plot, inset, show the median (center line), the upper and lower quartiles (the box), and the range of the data (the whiskers), excluding outliers.

a

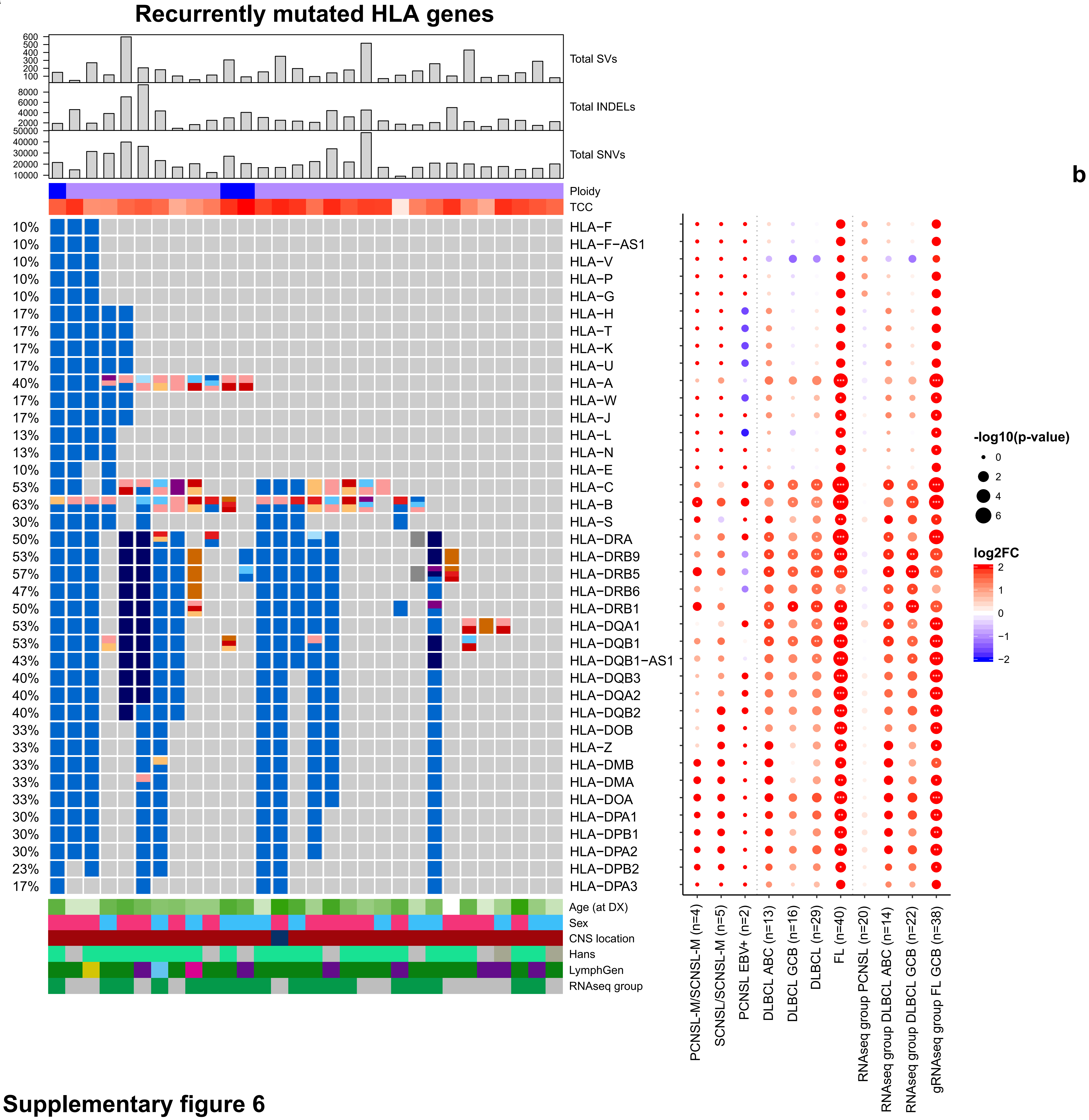

b

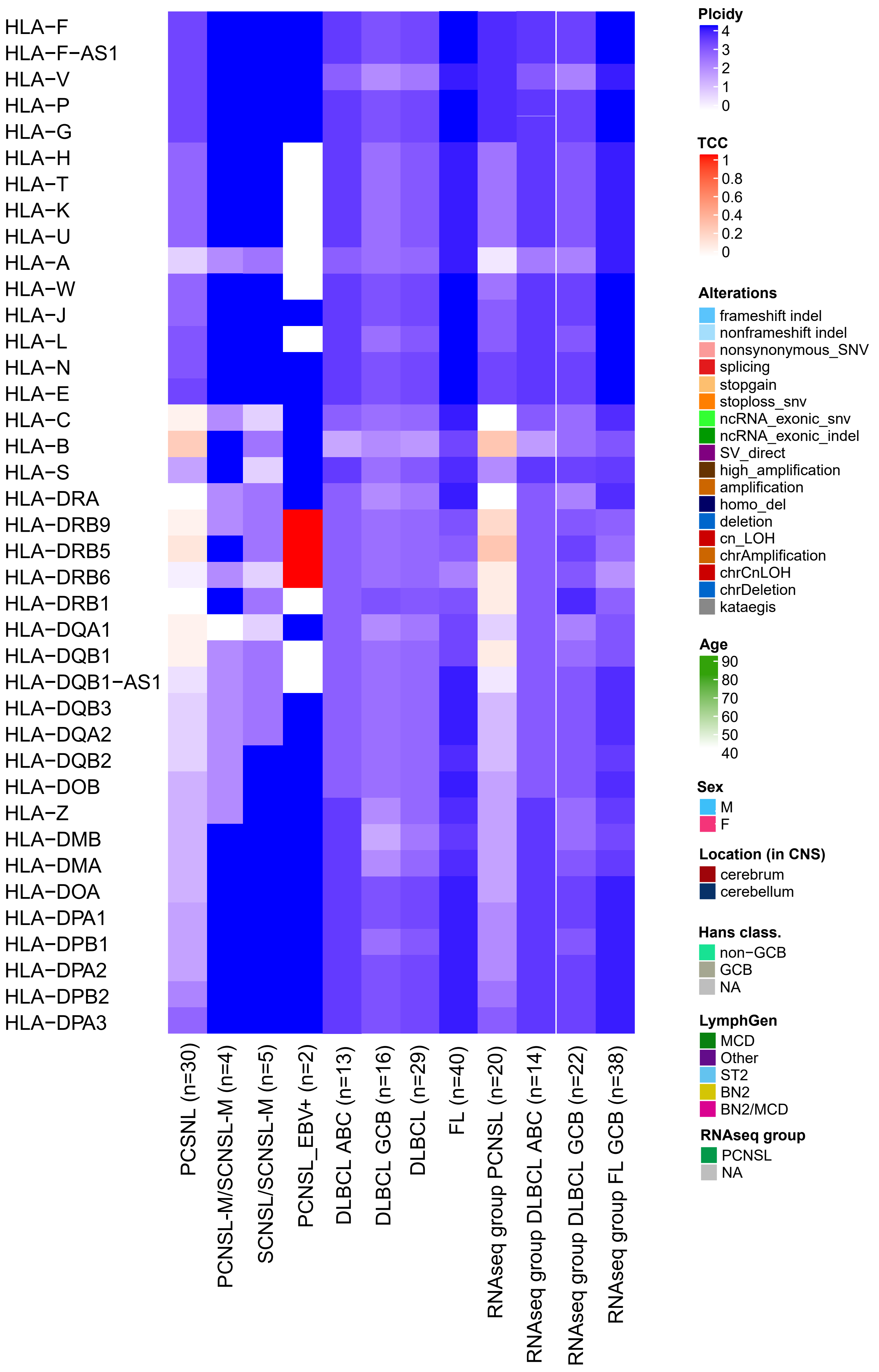

Supplementary figure 6

Supplementary figure 6: Mutational analysis of HLA loci.

(a) Oncoprint of recurrently mutated HLA genes in PCNSL. The top panel of the oncoprint shows the total numbers of structural variants (SVs), small insertions/deletions (INDELs), single nucleotide variants (SNVs), estimated ploidy, and tumour purity. Mutated genes are listed from top to bottom depending on their chromosome location. The colour of the box indicates the type of mutation. The corresponding dot plot reflects the log2 fold change and significance of alteration frequencies in the other subcohorts and RNAseq subgroups compared to PCNSL. The size of the dots demonstrate the significance according to a 2-tailed Fisher´s exact test. (b) The heatmaps reflect the alteration frequency of each gene in the other subcohort, RNAseq groups, and LymphGen groups.

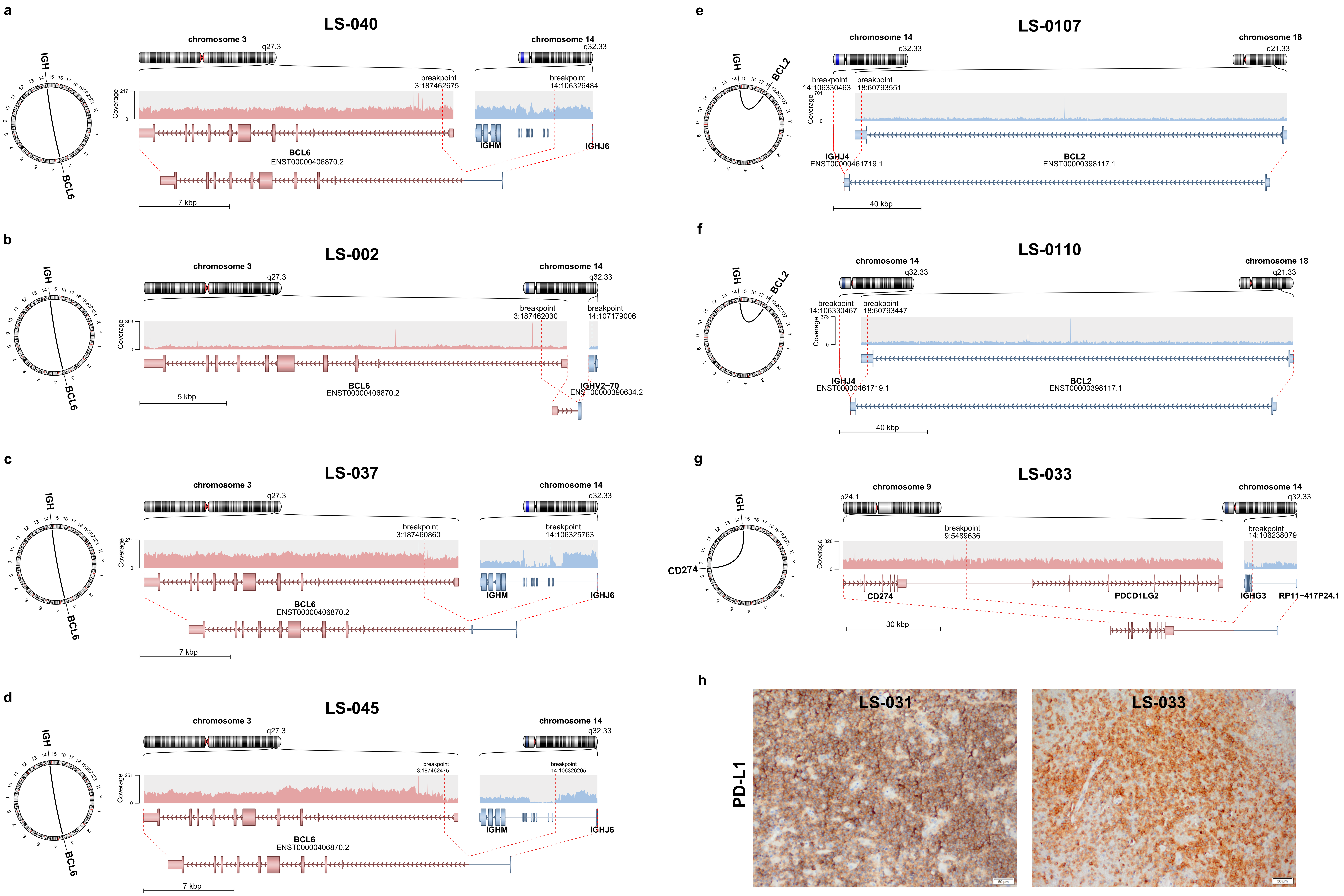

Supplementary figure 7

Supplementary figure 7: Analysis of immunoglobulin translocations breakpoints in PCNSL.

Schematic representation of the translocation breakpoints involving *BCL6* (PCNSL patients LS-040 (a), LS-002 (b), LS-037 (c), LS-045 (d)), *BCL2* (SCNSL patient LS-0107 (e), PCNSL-M patient LS-0110 (f)), and *CD274* (PD-L1; PCNSL patient LS-033, (g)). Immunohistochmical staining revealed PD-L1 expression in both PCNSL patients with translocations involving *CD274* (LS-031, LS-033 (h)).

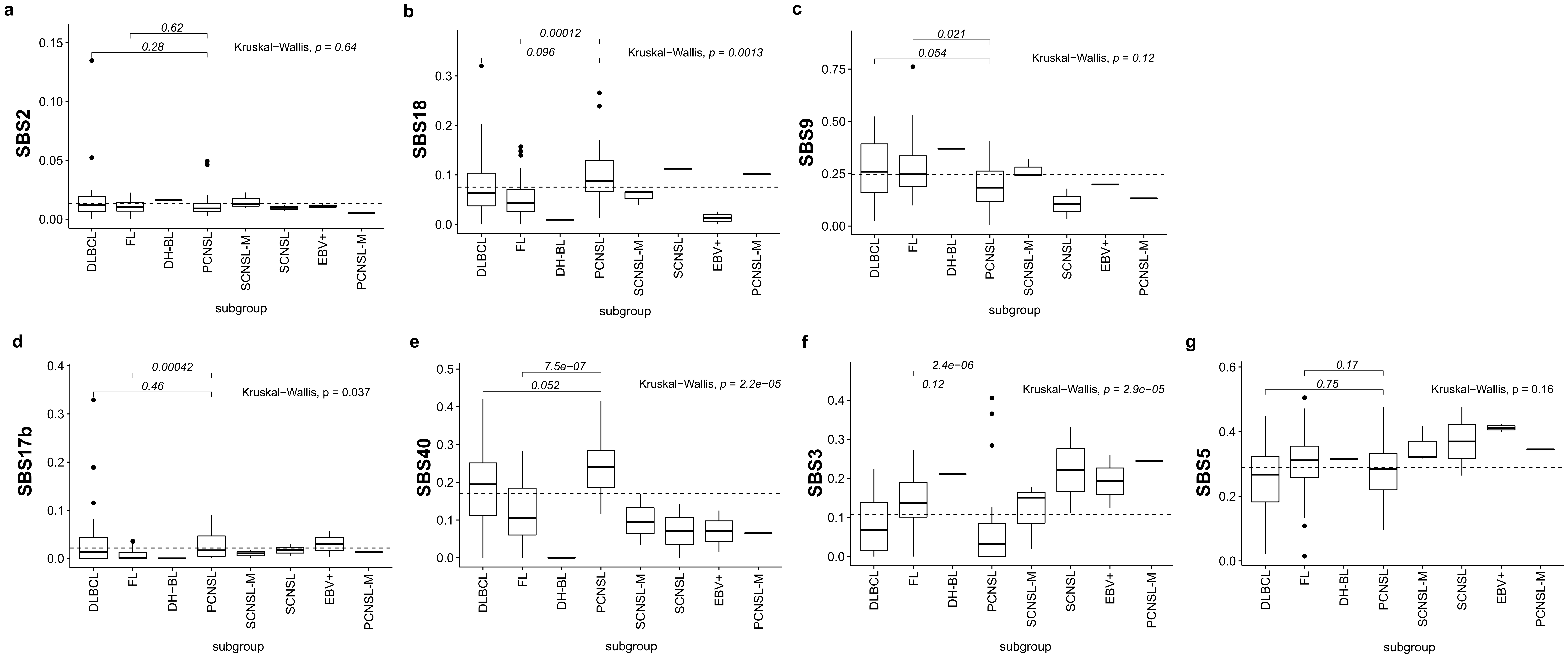

Supplementary figure 8

Supplementary figure 8: Analysis of single base substitutions (SBS).

Statistical analysis of SBS signatures in CNSL and peripheral lymphoma. Shown are the results of nonparametric Kruskal-Wallis H test and pairwise comparison of PCNSL with DLBCL or FL (one-sided Mann-Whitney U test, not corrected for multiple testing) for signatures SBS2 (a), SBS18 (b), SBS9 (c), SBS17b (d), SBS40 (e), SBS3 (f), and SBS5 (g). Box and whisker plots (a-g) show the median (center line), the upper and lower quartiles (the box), and the range of the data (the whiskers), excluding outliers.

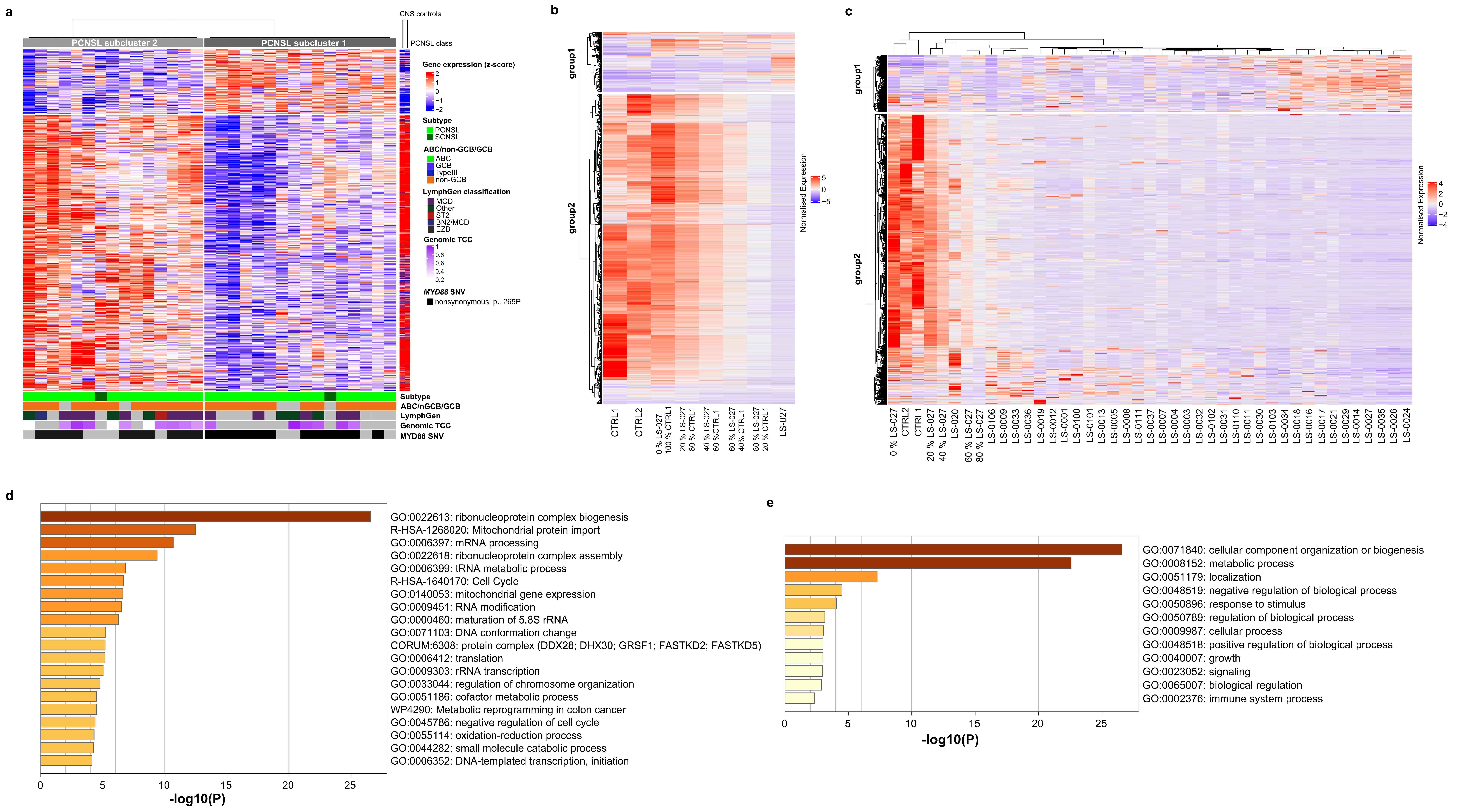

Supplementary figure 9

Supplementary figure 9: Transcriptomic signatures distinguish two PCNSL subcluster.

(a) RNA sequencing was performed using normal brain tissue (frontal lobe) controls (CTRL 1, 2) to extract the brain tissue signature from the PCNSL signature and to investigate the impact of normal brain tissue contamination in PCNSL samples. The heatmap shows unsupervised consensus clustering (using cola with "ATC" as top-value method), which revealed two groups: PCNSL1, ("pure", right) consisted of samples with high tumor cell content, and PCNSL2 ("impure", left) contained mainly samples with a lower tumor cell content, which signatures correlated well with normal brain tissue expression. (b) We mixed a pure PCNSL sample (LS-027) with increasing concentrations of RNA isolated from normal CNS tissue (CTRL 1, ranging from 0% to 80%). (c) The heatmap illustrate the impact of CNS tissue contamination in total RNA sequencing analysis of the tumour tissue. The differentially expressed genes of the pure PCNSL groups were analysed by Metascape[1] to identify the enriched pathways (d) and top three level Gene Ontology biological processes (e). Metascape adopts the hypergeometric test and employs the Benjamini-Hochberg correction for multiple testing.

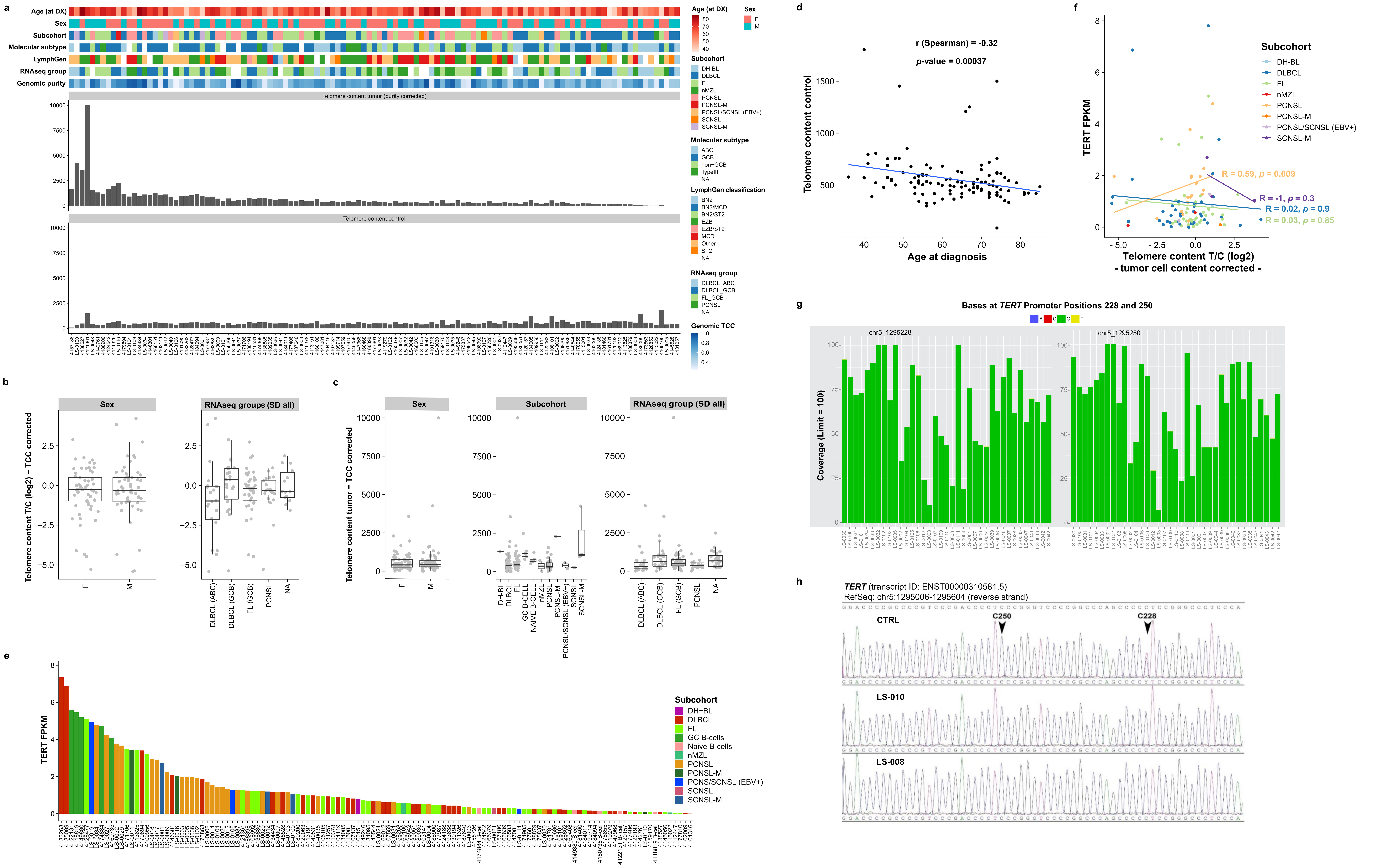

Supplementary figure 10

Supplementary figure 10: Analysis of *TERT* gene expression and telomere content in PCSNL.

(a) Telomere content in DLBCL, FL, PCNSL and SCNSL was estimated with TelomereHunter using default settings (filtering of telomere reads: at least six telomere repeats per 100 bp read length). In about 1/3 of the samples, the telomere content was higher in the tumor than in the control sample. Therefore, the telomere content of the blood control was used as an approximation for that of normal cells. (b) There was no statistical difference between PCNSL and systemic DLBCL in telomere content. Comparison of telomere content of different subgroups considering e.g. sex, subcohort, and RNAseq groups did not reach significance when corrected or (c) uncorrected for the control sample. Box and whisker plots in (b) and (c) show the median (center line), the upper and lower quartiles (the box), and the range of the data (the whiskers), excluding outliers. (d) We detected a negative correlation between age and the telomere content in the control samples (using the Pearson correlation-coefficient test without correction for multiple testing). (e) *TERT* expression in each sample of the CNSL and peripheral lymphoma cohort as well as in in the different subcohorts. (f) A significant positive correlation between *TERT* expression and telomere content was only observed in PCNSL. (g) We detected no *TERT* promotor mutation in the whole-genome sequencing cohort. The coverage was relatively high in most cases. If there was a mutation, the green bars would contain blue parts indicating the G>A mutations. (h) All WGS samples with coverage below 40 x (n = 6) and 31 additional FFPE CNSL samples (n = 21 PCNSL, n = 10 SCNSL) were analysed by Sanger sequencing. The Sanger sequencing chromatograms showing representative sequences of the *TERT* promoter region. A representative case of an Oligodendroglioma (positive control (CTRL)) showed a C228T mutation and a wild type C250. No *TERT* mutations have been detected in our cohort of CNSL patients (as exemplarily illustrated for patients LS-010 and LS-008).

Supplementary Reference

1. Zhou Y, Zhou B, Pache L, et al: Metascape provides a biologist-oriented resource for the analysis of systems-level datasets. Nat Commun 10(1): 1523; 2019.
