# Supplementary material for: The genomic and transcriptional landscape of primary central nervous system lymphoma
Source: Nat Commun. 2022 May 10;13:2558. doi: 10.1038/s41467-022-30050-y (PMC9091224; doi:10.1038/s41467-022-30050-y)
Supplement: Supplementary file 3 — Description of Additional Supplementary Files [file 41467_2022_30050_MOESM3_ESM.pdf]

Description of Additional Supplementary Files

File Name: Supplementary Data 1
Description: Study cohort.
The table gives an overview of important clinical, histological and molecular information of the CNSL and ICGC MMML samples investigated in this study. The CNSL subcohorts are highlighted in different colours.

File Name: Supplementary Data 2
Description: Whole-genome sequencing somatic mutation metrics.
The table gives an overview of the whole-genome sequencing somatic mutations metrics such as copy number alterations (CNAs), single nucleotide variants (SNVs), insertions and deletions (indels) and structural variations(SVs) for each sample.

File Name: Supplementary Data 3
Description: IntOGen cancer driver genes.
The table gives an overview of all driver genes identified by IntOGen in PCNSL. In addition to the IntOGen reported statistics, we report the fold change in mutational recurrence over the subgroups reported by Wright *et al* (LymphGen).

File Name: Supplementary Data 4
Description: Sanger sequencing results (WGS validation).
The table gives an overview of the bidirectional Sanger sequencing (bSS) results in the different CNSL subcohorts.

File Name: Supplementary Data 5
Description: Sanger sequencing results (Screening).
The table gives an overview of the bidirectional Sanger sequencing (bSS) results in the screening CNSL cohort.

File Name: Supplementary Data 6
Description: Mutual exclusivity of MYD88.
The table gives an overview of all mutations mutually exclusive with mutations in MYD88.

File Name: Supplementary Data 7
Description: Summary of mutational landscape.
The table gives an overview of 242 kataegis genes in PCNSL, excluding IG genes. These genes are compared to reported driver genes of subclasses from studies by other studies.

File Name: Supplementary Data 8
Description: Significant copy number alterations (CNAs).
The table gives an overview of significant CNAs identified by GITSIC2 in PCNSL in comparison to DLBCL and FL.

File Name: Supplementary Data 9
Description: Structural variations (SVs).
The table gives an overview of somatic SVs identified using SOPHIA in the different histological and molecular lymphoma subgroups.

File Name: Supplementary Data 10
Description: IG translocation partner.
The table gives an overview of IG translocation and putative partners predicted by SOPHIA in the CNSL cohort.

File Name: Supplementary Data 11
Description: IG translocations in CNSL cohort.
The table gives an overview of the genomic breakpoint positions of IG translocations according to hg19. The most probable mechanism leading to the IG translocations and the features detected at the breakpoint junction of the translocations are also described.

File Name: Supplementary Data 12
Description: RNA signature genes
The table gives an overview of the RNA signature genes in PCNSL, DLBCL and control samples.

File Name: Supplementary Data 13
Description: Recovered tumor in normal somatic mutations detected by TiNDA .
The table gives all mutations recovered by TiNDA in the CNSL cohort. Table format broadly resembles the VCF format, with additional columns for the patient ID (PID), variant allele fraction (VAF) in the tumor and control, and ANNOVAR annotation of region/function, and the closest/overlapping gene(s).

File Name: Supplementary Data 14
Description: Primer and antibodies.
The table lists all primers for Sanger sequencing and real-time PCR and gives information on the primary antibodies used for immunohistological stainings in the study.
