# Supplementary material for: The genomic and transcriptional landscape of primary central nervous system lymphoma
Source: Nat Commun. 2022 May 10;13:2558. doi: 10.1038/s41467-022-30050-y (PMC9091224; doi:10.1038/s41467-022-30050-y)
Supplement: Supplementary file 18 — Reporting Summary [file 41467_2022_30050_MOESM18_ESM.pdf]

nature research

Corresponding author(s): Josefine Radke, Naveed Ishaque

Last updated by author(s): Feb 17, 2022

Reporting Summary

Nature Research wishes to improve the reproducibility of the work that we publish. This form provides structure for consistency and transparency in reporting. For further information on Nature Research policies, see our Editorial Policies and the Editorial Policy Checklist.

Statistics

For all statistical analyses, confirm that the following items are present in the figure legend, table legend, main text, or Methods section.

n/a	Confirmed	
☐	☒	The exact sample size (*n*) for each experimental group/condition, given as a discrete number and unit of measurement
☐	☒	A statement on whether measurements were taken from distinct samples or whether the same sample was measured repeatedly
☐	☒	The statistical test(s) used AND whether they are one- or two-sided<br>*Only common tests should be described solely by name; describe more complex techniques in the Methods section.*
☐	☒	A description of all covariates tested
☐	☒	A description of any assumptions or corrections, such as tests of normality and adjustment for multiple comparisons
☐	☒	A full description of the statistical parameters including central tendency (e.g. means) or other basic estimates (e.g. regression coefficient) AND variation (e.g. standard deviation) or associated estimates of uncertainty (e.g. confidence intervals)
☐	☒	For null hypothesis testing, the test statistic (e.g. *F*, *t*, *r*) with confidence intervals, effect sizes, degrees of freedom and *P* value noted<br>*Give P values as exact values whenever suitable.*
☒	☐	For Bayesian analysis, information on the choice of priors and Markov chain Monte Carlo settings
☒	☐	For hierarchical and complex designs, identification of the appropriate level for tests and full reporting of outcomes
☐	☒	Estimates of effect sizes (e.g. Cohen's *d*, Pearson's *r*), indicating how they were calculated

Our web collection on statistics for biologists contains articles on many of the points above.

Software and code

Policy information about availability of computer code

Data collection	No software was used.
Data analysis	DNA sequencing read were aligned using the DKFZ AlignmentAndQCWorkflows v1.2.73. [https://github.com/DKFZ-ODCF/AlignmentAndQCWorkflows]. Reads were mapped to the human reference genome (build 37, version hs37d5) using bwa mem (version 0.7.8) with minimum base quality threshold set to zero [-T 0] and remaining settings left at default values, followed by coordinate sorting with biobambam bamsort (version 0.0.148) with compression option set to fast (1) and marking duplicate read pairs with biobambam bammarkduplicates with compression option set to best (9).<br>SNVs were called using the DKFZ SNVCallingWorkflow v1.2.166-1 [https://github.com/DKFZ-ODCF/SNVCallingWorkflow].<br>Indels were called, and Tumor-in-normal calculations were performed using the DKFZ IndelCallingWorkflow v1.2.177 [https://github.com/DKFZ-ODCF/IndelCallingWorkflow].<br>SV were called using the DKFZ SophiaWorkflow v1.2.16 [https://github.com/DKFZ-ODCF/SophiaWorkflow].<br>CNVs were called using DKFZ ACEseqWorkflow v1.2.8-4 [https://github.com/DKFZ-ODCF/ACEseqWorkflow].<br>Mutational signature analysis was performed using YAPSA development v3.13 using R v4.0.0.<br>Significant mutations were identified using IntOGen v3.0.8 using oncodriveclust v1.0.0, oncodrivefm v1.0.3, MutSigCV v 1.4, perl v5.20.3.1 (libraries perl-dbi v1.636, perl-digest-md5 v2.52, perl-digest-perl-md5 v1.9, perl-threaded v5.26.0), python v3.5.0 (libraries scipy v0.16.0, pycurl v7.43.0.3, numpy v1.10.0, pandas 0.17.0), Matlab Compiler Runtime v8.1 (2013a).<br>Mutual exclusivity analysis was performed using R v3.4.0 (library cometExactTest v0.1.5)<br>Significant CNV were identified using GISTIC v2.0.23 using MCR v83.<br>SV cohort plots were generated using perl v5.20.0, bedtools v2.24.0, R v3.3.1 (libraries circlize v0.4.5 and dplyr v0.7.8), using the gencode v19 gene models for annotation.<br>The mutations were integrated and plotted as oncoprint plots using using R v3.4.0 (library yapsa v3.13), perl v5.26.2 (libraries perl-getopt-long v2.50) and bedtools v2.16.2.<br>The telomere content was determined using TelomereHunter (v1.1.0) using python v3.5.6 (libraries pyyaml v3.13, pysam v0.9.1, pynacl

v1.2.1), samtools v1.3.1, bcftools v1.3.1 and htslib v1.3.2.
All WGS samples were classified into lymphoma subtypes using LymphGen v2.0 [https://llmpp.nih.gov/lymphgen/lymphgendataportal.php].
RNAseq reads were aligned using the DKFZ RNAseqWorkflow v1.2.22-6 [https://github.com/DKFZ-ODCF/RNAseqWorkflow], which uses STAR v2.5.2b, sambamba v0.6.5, samtools0.1.19 and featureCounts v1.5.1. Hierarchical consensus clustering was performed using cola v1.5.6.

For manuscripts utilizing custom algorithms or software that are central to the research but not yet described in published literature, software must be made available to editors and reviewers. We strongly encourage code deposition in a community repository (e.g. GitHub). See the Nature Research guidelines for submitting code & software for further information.

Data

Policy information about availability of data

All manuscripts must include a data availability statement. This statement should provide the following information, where applicable:

- Accession codes, unique identifiers, or web links for publicly available datasets
- A list of figures that have associated raw data
- A description of any restrictions on data availability

The raw sequencing data of the CNSL samples has been deposited at the European Genome-Phenome Archive (EGA) under the accession number EGAS00001005339 [https://ega-archive.org/studies/EGAS00001005339]. Access to the ICGC MMML-Seq raw sequencing data is available via the EGA under the accession number EGAS00001002199 [https://ega-archive.org/studies/EGAS00001002199] and EGAS00001001692 [https://www.ebi.ac.uk/ega/studies/EGAS00001001692].
Reference files used within in study include human reference genome (build 37, version hs37d5), Gencode v19 gene models, dbSNP10 build 141, and the 1000 Genomes Project phase 3 SNP database.

Raw image files of histological stainings, immunohistochemistry and FISH images generated in this study as well as all somatic mutation calls, integrated mutations tables and RNAseq counts on which the analysis was performed have been deposited at Zenodo [https://doi.org/10.5281/zenodo.6054242].

Sanger sequencing results are given in Supplementary data files 4 and 5. Raw data (ab1 files for Seq Software) has been edposited at the European Genome-Phenome Archive (EGA) under the accession number EGAS00001005339 [https://ega-archive.org/studies/EGAS00001005339].
EBV PCR: raw data (uncropped whole PCR gel) is given in the Supplementary Information file.
SYBR Green quantitative real-time PCR (qPCR) results of CDKN2A/B (exemplary from n=10 patients) is given in Supplement figure 1 e.
Raw real-time PCR data (original file (sds or eds file) has been edposited at the European Genome-Phenome Archive (EGA) under the accession number EGAS00001005339 [https://ega-archive.org/studies/EGAS00001005339].

Field-specific reporting

Please select the one below that is the best fit for your research. If you are not sure, read the appropriate sections before making your selection.

☒ Life sciences ☐ Behavioural & social sciences ☐ Ecological, evolutionary & environmental sciences

For a reference copy of the document with all sections, see nature.com/documents/nr-reporting-summary-flat.pdf

Life sciences study design

All studies must disclose on these points even when the disclosure is negative.

Sample size

We enrolled 51 patients with central nervous system lymphomas (CNSL) for whole-genome (WGS, n = 38) and RNA sequencing (RNAseq, n = 37) analysis, including n = 24 samples subjected to both workflows. All samples were classified as DLBCL. No statistical methods were used to predetermine sample sizes. We included all individuals (starting in 2013) with DLBCL of the CNS where sufficient material was available as specified in the description of study design. This study is - to our knowledge - the largest cohort of PCNSL to date.

Additionally, n=2 normal brain tissue controls were enrolled for RNA sequencing.

For comparison, we used and reanalyzed an early release of meanwhile published whole-genome and RNA sequencing data obtained by the ICGC MMML-Seq Consortium from systemic diffuse large B-cell lymphoma (DLBCL, total: n = 36, WGS: n = 29, RNAseq: n = 36, both workflows: n = 29), follicular lymphoma (FL, total: n = 39, WGS: n = 39, RNAseq: n = 38, both workflows: n = 38), and one “double hit” (DH)-lymphoma with a molecular BL signature. Additionally, we included WGS and RNAseq data from a single EBV-PCNSL case as well as RNAseq data from two nodal marginal zone lymphomas (nMZL) as well as naïve (n = 5) and GC B-cells (n = 5) as normal controls. These data were obtained by the ICGC MMML-Seq consortium in accordance to protocols previously published.

Data exclusions

We only included CNSL, which were classified as DLBCL. No data were excluded from the analyses.

Replication

Histological diagnosis of lymphoma samples was performed by at least two consultants of (neuro)pathology with agreement in Heidelberg and Berlin. Histological stainings were replicated at least once with the appropriate positive and negative controls. Each replication was successful.
Immunohistochemistry and FISH analyses were technically replicated at least once. Each replication was successful.
Recurrent point mutations found in WGS were validated by Sanger sequencing. Sanger sequencing was replicated once if a result was unclear. If the results was unclear in the second repetition it was listed as NA. Additionally, recurrent point mutations were validated in an independent series of CNSL (screening cohort) by Sanger sequencing. Again the Sanger sequencing was replicated in case the results were unclear.

Randomization	Randomization is not relevant for this study as we included all patients with DLBCL within the CNS where sufficient material was available as described in the study design.
Blinding	Blinding was implemented through use of unsupervised analysis.<br>Whole-genome sequencing, RNA-sequencing, Sanger sequencing, real-time PCR, immunohistological stainings, and FISH were performed in a blinded fashion.

Reporting for specific materials, systems and methods

We require information from authors about some types of materials, experimental systems and methods used in many studies. Here, indicate whether each material, system or method listed is relevant to your study. If you are not sure if a list item applies to your research, read the appropriate section before selecting a response.

Materials & experimental systems

n/a	Involved in the study	
☐	☒	Antibodies
☒	☐	Eukaryotic cell lines
☒	☐	Palaeontology and archaeology
☒	☐	Animals and other organisms
☐	☒	Human research participants
☒	☐	Clinical data
☒	☐	Dual use research of concern

Methods

n/a	Involved in the study	
☒	☐	ChIP-seq
☒	☐	Flow cytometry
☒	☐	MRI-based neuroimaging

Antibodies

Antibodies used

The following primary antibodies were used:
Mouse Anti-Human Bcl-6 (dilution: 1:10; company: DAKO; clone: PG-B6p; host: mouse; catalogue number: M7211; LOT: 20036842)
Mouse Anti-Human CD10 (dilution: 1:10; company: Novocastra; clone: 56C6; host: mouse; catalogue number: NCL-CD10-270; LOT: 6084280)
Mouse Anti-Human CD20 (dilution: 1:400; company: DAKO, clone: L26; host: mouse; catalogue number: M0755; LOT: 20074170)
Rabbit Anti-Human CD3 (dilution: 1:100; company: DAKO; clone: - ; host: rabbit; catalogue number: A0452; LOT: 20061852)
Mouse Anti-Human CD45 (dilution: 1:400; company: DAKO; clone: 2B11 + PD7/26; host: mouse; catalogue number: M0701; LOT: 20083127)
Mouse Anti-Human CD79a (dilution: 1:100; company: DAKO; clone: JCB117; host: mouse; catalogue number:M7051; LOT: 20030096)
Mouse Anti-Human EBV-LMP1 (dilution: 1:1000; company: DAKO; clone: CS1-4; host: mouse; catalogue number M0897; LOT: 00082511)
Mouse Anti-Human Ki67 (dilution: 1:100; company: DAKO; clone: Mib-1; host: mouse; catalogue: M7240; LOT: 41315040)
Mouse Anti-Human MUM1 (dilution: 1:50; company: DAKO; clone: MuM1P; host: mouse; catalogue number: M7259; LOT: 41255196)
Mouse Anti-Human PD-L1 (dilution: 1:200; company: Cell Siganling; clone: - ; host: mouse; catalogue number: 13684; LOT: 13)

Immunohistochmical stainings were performed using an automated platform (VENTANA™). Primary antibodies were applied and developed using the iVIEW DAB Detection Kit (Ventana Medical Systems; Catalog Number: 760-091)

Validation

All antibodies used in this study are validated for the daily routine diagnostic workflow of the institutes of Neuropathology and of Pathology (Charité) and have been widely used in several studies (please see citations below). All antibodies were validated for the use of immunohistochemistry (IHC) by the manufacturer. Positive controls (tissue known to express the protein of interest) were either integrated as on-slide controls or as separate slide within the same automated IHC run. The following positive (PC) and negative controls (NC) are used:

Bcl-6 (PC: tonsil (germinal center); NC: tonsil (T-cell zone))
References for BCL-6:
Flenghi L, Bigerna B, Fizzotti M, Venturi S, Pasqualucci L, Pileri S, et al. Monoclonal antibodies PG-B6a and PG-B6p recognize, respectively, a highly conserved and a formol-resistant epitope on the human BCL-6 protein amino-terminal region. Am J Pathol 1996,148:1543-55.
Falini B, Bigerna B, Pasqualucci L, Fizzotti M, Martelli MF, Pileri S, et al. Distinctive expression pattern of the BCL-6 protein in nodular lymphocyte predominance Hodgkin's disease. Blood 1996,87:465-71.

CD10 (PC: tonsil (germinal center); NC: tonsil (T-cell zone))
References for CD10:
Chu PG, Chang KL, Weiss LM et al. Immunohistochemical detection of CD10 in paraffin sections of hematopoietic neoplasms: a comparison with flow cytometry detection in 56 cases. Applied Immunohistochemistry & Molecular Morphology 2000 8(4), 257–262.
Conde-Sterling DA, Aguilera NS, Nandedkar MA et al. Immunoperoxidase detection of CD10 in Precursor T-lymphoblastic lymphoma/leukemia: a clinicopathologic study of 24 cases. Archives of Pathology & Laboratory Medicine 2000 124(5), 704-708.
Endoh Y, Tamura G, Motoyama T et al. Well-differentiated adenocarcinoma mimicking complete-type intestinal metaplasia in the stomach. Human Pathology 1999 30(7), 826-832.
Kaufmann O, Flath B, Späth-Schwalbe E et al. Immunohistochemical detection of CD10 with monoclonal antibody 56C6 on paraffin sections. American Journal of Clinical Pathology 1999 111(1), 117-122.
McIntosh GG, Lodge AJ, Watson P et al. NCL-CD10-270: a new monoclonal antibody recognising CD10 in paraffin-embedded tissue.

American Journal of Pathology 1999 154(1), 77–82.
Millar E K, Waldron S, Spencer A et al. CD10 positive thyroid marginal zone non-Hodgkin lymphoma. Journal of Clinical Pathology 1999 52, 849-850.

CD20 (PC: tonsil (germinal center); NC: tonsil (T-cell zone))
References for CD20:
Lesch B, Tothova Z, Morgan E, Liao Z, Bronson R, Ebert B, et al. Intergenerational epigenetic inheritance of cancer susceptibility in mammals. elife. 2019;8:
Rao D, Gurish M, Marshall J, Slowikowski K, Fonseka C, Liu Y, et al. Pathologically expanded peripheral T helper cell subset drives B cells in rheumatoid arthritis. Nature. 2017;542:110-114

CD3 (PC: tonsil (T-cell zone): DAKO; NC: tonsil (germinal center))
Cassidy L, Young A, Young C, Soilleux E, Fielder E, Weigand B, et al. Temporal inhibition of autophagy reveals segmental reversal of ageing with increased cancer risk. Nat Commun. 2020;11:307
Harel M, Ortenberg R, Varanasi S, Mangalhara K, Mardamshina M, Markovits E, et al. Proteomics of Melanoma Response to Immunotherapy Reveals Mitochondrial Dependence. Cell. 2019;179:236-250.e18

CD45 (PC: skin-associated lymphoid tissue; NC: epithelium, epidermis)
References for CD45:
Crippa S, Rossella V, Aprile A, Silvestri L, Rivis S, Scaramuzza S, et al. Bone marrow stromal cells from β-thalassemia patients have impaired hematopoietic supportive capacity. J Clin Invest. 2019;129:1566-1580
inchen J, Chen H, Parikh K, Antanaviciute A, Jagielowicz M, Fawkner Corbett D, et al. Structural Remodeling of the Human Colonic Mesenchyme in Inflammatory Bowel Disease. Cell. 2018;175:372-386.e17

CD79a (PC: tonsil (germinal center); NC: tonsil (T-cell zone))
References for CD79a:
Mason DY, Cordell JL, Brown MH, Borst J, Jones M, Pulford K, et al. CD79a: a novel marker for B-cell neoplasms in routinely processed tissue samples. Blood 1995,86:1453-9.
Pilozzi E, Pulford K, Jones M, Muller-Hermelink HK, Falini B, Ralfkiaer E, et al. Co-expression of CD79a (JCB117) and CD3 by lymphoblastic lymphoma. J Pathol 1998,186:140-3.
Chu PG, Arber DA. CD79: a review. Appl Immunohistochem Mol Morphol 2001,9:97-106

EBV-LMP1 (PC: case of Epstein Barr virus-related lymphoproliferative disorder (EBV-PTLD); NC: normal brain tissue)
References for EBV-LMP1:
Izawa K, Martin E, Soudais C, Bruneau J, Boutboul D, Rodriguez R, et al. Inherited CD70 deficiency in humans reveals a critical role for the CD70-CD27 pathway in immunity to Epstein-Barr virus infection. J Exp Med. 2017;214:73-89
Ikeda J, Wada N, Nojima S, Tahara S, Tsuruta Y, Oya K, et al. ID1 upregulation and FoxO3a downregulation by Epstein-Barr virus-encoded LMP1 in Hodgkin's lymphoma. Mol Clin Oncol. 2016;5:562-566

Ki67 (PC: tonsil (germinal center); NC: tonsil (T-cell zone))
References for Ki67:
Zhu X, Chen L, Huang B, Wang Y, Ji L, Wu J, et al. The prognostic and predictive potential of Ki-67 in triple-negative breast cancer. Sci Rep. 2020;10:225
Oyama Y, Nishida H, Kusaba T, Kadowaki H, Arakane M, Okamoto K, et al. Colon adenoma and adenocarcinoma with clear cell components - two case reports. Diagn Pathol. 2019;14:37

MUM1 (PC: tonsil (germinal center); NC: tonsil (T-cell zone))
References for MUM1:
Shimono J, Miyoshi H, Kamimura T, Eto T, Miyagishima T, Sasaki Y, et al. Clinicopathological features of primary splenic follicular lymphoma. Ann Hematol. 2017;96:2063-2070
Banat G, Tretyn A, Pullamsetti S, Wilhelm J, Weigert A, Olesch C, et al. Immune and Inflammatory Cell Composition of Human Lung Cancer Stroma. PLoS ONE. 2015;10:e0139073

PD-L1 (PC: case of lung metastasis; NC: vessels endothelium with the same sample)
References for PD-L1:
Sorrentino C, D'Antonio L, Fieni C, Ciummo SL, Di Carlo E. Colorectal Cancer-Associated Immune Exhaustion Involves T and B Lymphocytes and Conventional NK Cells and Correlates With a Shorter Overall Survival. Front Immunol. 2021.
Kumazawa T, Mori Y, Sato H, Permata TBM, Uchihara Y, Noda SE, Okada K, Kakoti S, Suzuki K, Ikota H, Yokoo H, Gondhowiardjo S, Nakano T, Ohno T, Shibata A. Expression of non-homologous end joining factor, Ku80, is negatively correlated with PD-L1 expression in cancer cells after X-ray irradiation. Oncol Lett. 2022

Standardized validation proceedure for all antibodies used in this study:
All antibodies were checked for reproducability and integrity of the assay three times in independent staining experiments and in at least three different positive samples and compared to expected staining patterns in the controls regarding published expression of the antigens were applicable. Furthermore an additional secondary-only antibody control (i.e. omission of first antibody) was performed for every setup.

Human research participants

Policy information about studies involving human research participants

Population characteristics

Fresh frozen and paraffin embedded primary central nervous system lymphoma (PCNSL) and secondary central nervous system lymphoma (SCNSL) tumor tissue and matching blood samples (germline control) were acquired from the Department of Neuropathology, Charité, Berlin (Germany), and the Department of Neurosurgery, Heidelberg (Germany) from

	chemotherapy-naïve, adult (age: ≥18) patients. We enrolled CNSL samples from 51 adults diagnosed with PCNSL or SCNSL. Median age was 69, mean age was 66.5 years at diagnosis (range 40-82 years). The female:male ratio was 1.3:1.
Recruitment	Not applicable. We enrolled all adult patients with DLBCL within the CNS, where sufficient material was available.
Ethics oversight	This study was approved by the Ethics Committee of the Charité (EA1/245/13) and was in compliance with the Declaration of Helsinki. Informed consent was obtained from all participants in the study.

Note that full information on the approval of the study protocol must also be provided in the manuscript.
